# Antibiotic Use in European Pig Production: Less Is More

**DOI:** 10.3390/antibiotics11111493

**Published:** 2022-10-27

**Authors:** Jeroen Dewulf, Philip Joosten, Ilias Chantziaras, Elise Bernaerdt, Wannes Vanderhaeghen, Merel Postma, Dominiek Maes

**Affiliations:** 1 Veterinary Epidemiology Unit, Faculty of Veterinary Medicine, Ghent University, Salisburylaan 133, 9820 Merelbeke, Belgium; 2 Unit of Porcine Health Management, Faculty of Veterinary Medicine, Ghent University, Salisburylaan 133, 9820 Merelbeke, Belgium; 3 Centre of Expertise on Antimicrobial Consumption and Resistance in Animals (AMCRA), Galileolaan 5/02, 1210 Brussels, Belgium

**Keywords:** antibiotic use, antimicrobial use, pigs

## Abstract

The goal of this study is to describe the current use of antibiotics in the European pig industry based on an extensive literature review. To achieve this, an overview of results from national (*n* = 15) and multi-country (*n* = 2) cross-sectional and longitudinal (*n* = 2) surveys, which describe antimicrobial use in pigs, is presented. Results are further linked to the outcome of the European Surveillance of Veterinary Antimicrobial Consumption (ESVAC) project. Overall, it was found that weaned piglets received the most antibiotics, followed by suckling piglets resulting in over 80% of the treatments being administered to animals before 10 weeks of age. Furthermore, it was observed that antibiotic use (ABU) was significantly associated across age categories, indicating that farms with a high use in piglets also used more antibiotics in their finishers. This may, among other things, be explained by farmers’ habits and behavior. However, above all, the studies showed surprisingly large differences in ABU between the countries. These differences may be related to the differences in disease prevalence and/or differences in the level of biosecurity. However, they may also reflect variations in rules and regulations between countries and/or a difference in attitude towards ABU of farmers and veterinarians that are not necessarily linked to the true animal health situation. Furthermore, it was observed that already a substantial proportion of the European pig production is able to successfully raise pigs without any group treatments, indicating that it is possible to rear pigs without systematic use of antibiotics. Based on the ESVAC data, a decline of 43.2% was observed in sales of antibiotics for animals in Europe between 2011 and 2020. To enable efficient antimicrobial quantification and stewardship, 15 European countries have already established systems for herd level monitoring ABU in pigs.

## 1. Introduction

In the present paper, the authors use the term “antibiotic” use or resistance instead of “antimicrobial”, the latter refers to drugs or resistance for any type of microbial organism, such as bacteria, viruses, or parasites. Antibacterial use and resistance would hence be a more correct description, though it is hardly ever used. Since antibiotics is the everyday word for antibacterial products, the term antibiotic resistance has become generally accepted and is therefore used in this paper. For consistency, the term “responsible use” of antibiotics will be used throughout the paper, although judicious or prudent use of antimicrobials might also be used in the literature.

Antibiotics had their first applications in humans, revolutionizing the practice of medicine and gaining early on the nickname “miracle drugs”. In the nineteenth century, almost half of all deaths were related to infectious diseases in England and Wales. At the dawn of the 21st century, the burden of infectious diseases had become relatively limited compared to non-infectious causes [1]. Moreover, antibiotics have played an important role in catalyzing for medical improvements. Without them, many achievements in modern medicine, such as major surgeries, organ transplantation, or cancer chemotherapy, would become impossible, as they would imply the same high risks due to infections as in pre-antimicrobial times [2,3]. Although antibiotics were considered to be miracle drugs in the early days of discovery, bacteria are resilient in that they adapt and can change rapidly in response to changes in their environment, such as the presence of an antibiotic, in an effort to survive they develop resistance. As such, the selection towards resistant bacteria is an adaptation of the microorganism to its environment. Although the epidemiology of antibiotic resistance selection and spread may be complex, the association between antibiotic use and antibiotic resistance has been described numerous times [4]. 

The discovery of antibiotics also had their impact on veterinary medicine, adding to a better health and welfare in animals [5]. Penicillin had its first application in dairy cows, where it was used to treat mastitis [6]. Treatments like the latter can be administered individually, which is mostly the case in companion animals, dairy cattle, horses, and breeding pigs. However, when larger groups of animals need to be treated such as in poultry or swine, group treatments are often provided orally via feed or water [7,8]. When such a group medication is initiated because of clinical signs in only a subgroup of the animals, this is called a metaphylactic treatment. When it is administered to prevent problems at critical stages in the production phase (e.g., at weaning), in groups of animals not showing clinical signs, these administrations are prophylactic. The discovery and early use of antibiotics in animal production coincided with the post-war revolution from small scale extensive livestock production systems to a more intensive industrialized livestock production, where animals were housed indoor in large flocks and herds [9,10]. This evolution not only resulted in farmers using more metaphylactic and prophylactic medication, it also paved the way for the commercial application of antibiotics as growth promotor. 

Obviously, the use of antibiotics in animals also resulted in the selection of resistant bacteria in animals which was recently clearly documented in an EFSA study on swine diseases caused by bacteria resistant to antibiotics [11]. To what extent antibiotic use and resistance in animals also influence the occurrence of resistance in humans remains an important area of debate. Although some studies suggested to provide evidence that transmission of antibiotic resistance from food animals to humans may occur, a recent literature review reported that no firm conclusions can be drawn on the directionality of transmission due to limitations in study methodologies [12].

## 2. The Ban on Growth Promotors in Europe

Upon their introduction, antibiotics were, aside from their therapeutic applications, quickly associated with a higher production in livestock farming [13], earning them the nickname “antimicrobial growth promotors” (AGPs). In these early days several studies showed a considerable benefit of AGP with productivity gains ranging from 1% to double digits, depending on factors such as nutrition, breeding, housing, etc. [13,14,15]. However, in present farm settings where modern production techniques and management practices are adhered to, this “benefit” is shown to be lower if not negligible [16]. Hence, AGPs should have no place in modern animal production as they promote antibiotic resistance. In response, several countries introduced legislation acts to gradually phase out its use. In Europe, one of the first countries to introduce such measures was Sweden [17]. Although the AGPs ban in Sweden did lead to some initial animal health problems, this was successfully addressed by improved management and disease prevention [17]. In line with the positive feedback from Sweden and other countries, EC decided to ban the use of AGPs totally from 2006 onwards. In contrast to some expectations, this did not result in a substantial decline in food animal production in Europe. In Denmark and the Netherlands, a shift towards increased therapeutic antibiotic use (ABU) was also observed after the ban of AGPs [18]. However, this increase turned out to be only temporary and was even non-existent in other countries such as Norway [19,20].

## 3. Systematic Monitoring of Antibiotic Sales Data in Animals in Europe

**Figure 1:**
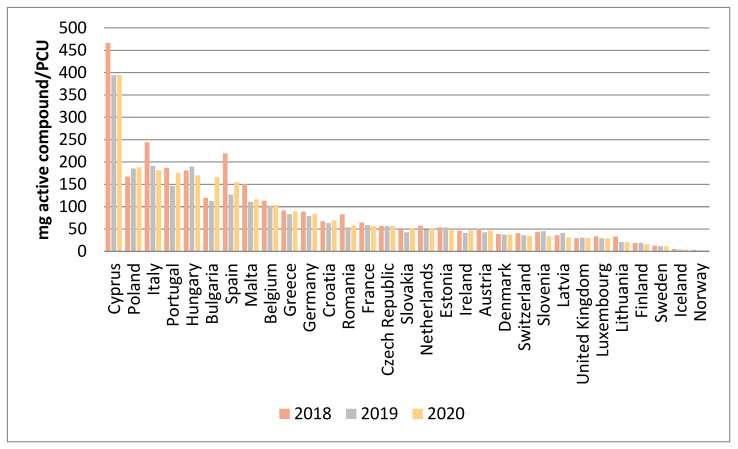
Antimicrobial use in animals in 31 European countries expressed in mg active component/ PCU between 2018 and 2020 (based on eleventh ESVAC report, 2021).

Already in 2005, the European Medicines Agency (EMA) launched the European Surveillance of Veterinary Antimicrobial Consumption (ESVAC) project. In this project, ABU in animals in Europe is being quantified at country level based on national sales data of veterinary antibiotics. The last report (data up to 2020) provides information on the sales of antibiotics in 31 European countries [21] (Figure 1). The use is expressed in milligrams of active component sold in relation to the Population Correction Unit (PCU), which is a proxy for the biomass of the food-producing animal population (including horses) in a country. 

The ESVAC data provide a comprehensive and recurrent overview of the total sales of antibiotics in Europe. The results clearly show huge differences between countries with the highest and lowest sales ranging from 393.9 mg/PCU to 2.3 mg/PCU and a median value of 51.9 mg/PCU. Although part of the variation can be explained by differences in the demography of the animal population, also animal health and human behavior factors are likely influencing these outcomes. For the 25 countries which provided sales data for all years between 2011 and 2020, an overall decline in sales (mg/PCU) of 43.2% was observed, with a noticeable decrease in sales identified for some of the highest-scoring countries.

Although these ESVAC data are successful in monitoring the overall trends in ABU in animals in Europe, it is difficult to extract the specific evolution in ABU for specific types of animals (e.g., pigs) due to the fact that there are many antibiotics on the market that are registered for multiple species, and therefore they cannot easily be assigned to one specific animal species. Nonetheless, in several European countries, pig production accounts for a major part of the animal production and it can therefore safely be assumed that the observed reductions in ABU are partially due to reductions of ABU in pig production. Most likely, these reductions have been achieved by implementing proper biosecurity measures and farm health management practices [22,23,24].

## 4. Quantitative Insights in Antimicrobial Use in Pigs in Europe

### 4.1. Surveys on Antibiotic Use in Pigs in Europe

Since the ban on AGPs in Europe, the focus has shifted to therapeutic, metaphylactic, and prophylactic use of antibiotics in animals. This has resulted in a number of surveys in European countries describing ABU in pigs, both quantitatively and qualitatively. These surveys were conducted in Belgium [8,25], Denmark [11,26], Spain [27], Germany [28,29], Sweden [30], France [31], Italy [32,33], Ireland [34], Finland [35,36], and Switzerland [37].

These surveys have been a crucial step towards a more detailed understanding of ABU and its risk factors in pig production. Many of these studies described huge differences in ABU over the course of the production cycle, with the majority of the use in young pigs. Other typical findings include a large variation in ABU between farms within the same country and the frequent application of prophylactic medication, often with important contributions of critically important antibiotics such as 3rd generation cephalosporins and fluoroquinolones. In addition, increased farm size, veterinarian, poor biosecurity, and farm health management have all been described as drivers for ABU. Some of the studies also were longitudinal in nature and provided evidence for the possibility of reducing ABU over time, without necessarily jeopardizing animal health and productivity [38,39]. This was in contrast to a common fear among farmers and veterinarians, namely that high ABU would be necessary to support intensive production and that reducing ABU would result in lower production outputs. 

A limitation of the described surveys is the huge variation in metrics used to quantify antimicrobial use, hampering comparisons between countries [38,40]. Therefore, the execution of multi-country studies, using the same quantification methodologies was a big step forward. A first multi-country study, including four countries, namely France, Germany, Sweden, and Belgium, was described by Sjölund et al. [41]. In this study, it was found that weaned piglets received the most antibiotics, followed by suckling piglets. Furthermore, it was observed that ABU was significantly associated across age categories, indicating that farms with a high use in piglets also used more antibiotics in their finishers. This may, among other things, be explained by farmers’ habits and behavior [42]. However, above all, the study showed surprisingly large differences in ABU between the countries included. These differences between countries, but also between herds, might be related to the differences in disease prevalence [43,44] and/or differences in the level of biosecurity [24,45]. However, they may also reflect variations in the attitude towards ABU of farmers and veterinarians, which are not necessarily linked to the true animal health situation. Several studies have shown that there is a link between the attitude towards antibiotics and antibiotic use [22,46,47]. 

**Figure 2:**
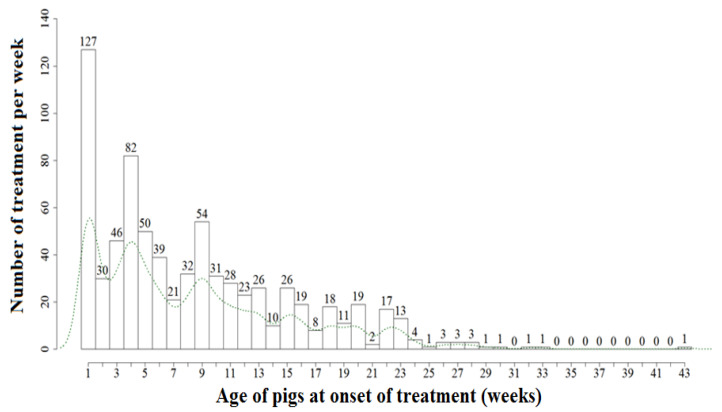
Histogram showing the number of antimicrobial group treatments per week applied to a batch of pigs from birth to slaughter based on 750 treatments (30 instances of treatments missing). The green dotted line represents the weekly average treatment incidence (adapted from Sarrazin et al., 2019) [48].

Responding to the challenge of collecting good quality ABU data on farm level, a study involving nine European countries was set to follow all antibiotic use administered within a single batch for a total of 180 pig farms [48]. In this study, ABU was quantified using the treatment incidence (TI) indicator based on the defined daily doses for animals (DDDvet) as provided by the European Medicines Agency (EMA). TIDDDvet represents the percentage of time a pig is treated with antibiotics in a defined period [25]. Although a multitude of systems are available to quantify antibiotic use [49,50], the use of the TI methodology, reflecting the number of treatment days out of 100 days present on the farm, has multiple advantages for ABU data collection at farm level: it corrects for differences in molecular weight of different antibiotics, it takes into account the size/weight of the population at risk for treatment, it corrects for the long acting molecules, and it expresses the ABU in a unit that is easy to understand and communicate. The main limitation of the methodology is that it requires quite detailed data collection. Nonetheless, this methodology has been put forward in the AACTING (network on quantification of veterinary antimicrobial usage at herd level and analysis, communication, and benchmarking to improve responsible use) guidelines with the aim of assisting parties in setting up systems for monitoring of farm-level ABU [50]. The results of the multi-country study in 9 European countries revealed that the majority of antibiotics were administered to weaners (69.5% of total TIDDDvet) followed by suckling piglets (22.5% of total TIDDDvet) (Figure 2). 

**Figure 3:**
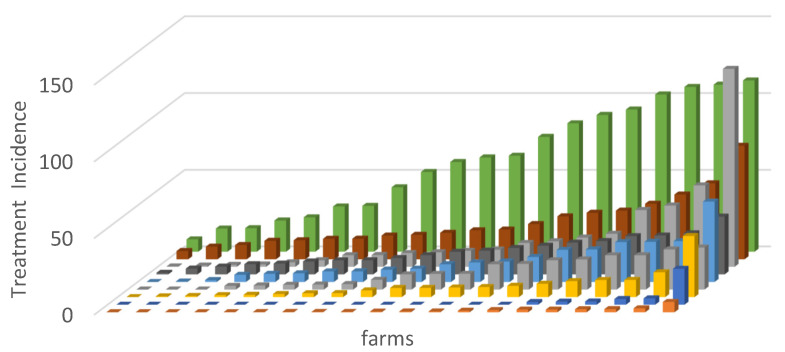
Country-level comparison of TI per 100 pig-days at risk, based upon group treatment data for a standardized lifespan of 200 days based on defined daily doses for animals (TI200_DDDvet_). Every color represents a different European country included in the study (9 countries). Every beam represents a farm (20 farms per country) (adapted from Sarrazin et al., 2019) [48].

In this multi-country study, ABU varied again considerably between countries and farms with a median TIDDDvet of 9.2 for a standardized rearing period of 200 days (Figure 3). This means that the median pig in this study received antibiotics during 9.2% of its lifetime from birth to slaughter, or approximately 18 days in total [48]. Noteworthy is the fact that 11.7% of the farms did not use any group treatments of antibiotics throughout the production round. Yet, the large variation in ABU patterns (choice and/or amount of antibiotics used) between farms revealed the need to take action towards a more responsible ABU such as a reduced ABU at strategic time points in combination with increased biosecurity. In addition, the selection of the used molecules could change away from the use of use of the highest priority critical important antibiotics such as colistin, fluoroquinolones and 3rd and 4th generation cephalosporins. 

Extended spectrum penicillins (31.2%) and polymyxins (24.7%) were the active substances most often used in group treatments in this multi-country study, with the majority administered through feed or water (82%), although considerable differences were observed regarding the administration route between countries. In one country, 59% of all treatments were parenterally, whereas in another country this was only 7%. The average treatment duration for parenterally administered antibiotics was 2.6 days (minimum 1; median 3; maximum 14) for non-long acting (LA) formulations and 5.2 days for LA formulations, while for oral treatments, this was 10.6 days on average. Moreover, 10% of the oral treatments were applied for a period of at least 21 consecutive days. In agreement with the study of Sjölund et al. [41], it was found that higher ABU at a young age was associated with higher use in older pigs. The most frequent indication for treatment was the category “general” (37.5%) which clearly reveals the need for a better diagnosis. Other indications for treatment were intestinal (24.4%) and respiratory disorders (20.1%) [48].

### 4.2. Herd Level Monitoring of Use Data

When moving from sales data to use data, typically herd level data collection systems are needed. In Europe, several countries have already established or are developing systems for monitoring ABU in pigs. Currently, AACTING (network on quantification of veterinary antimicrobial usage at herd level and analysis, communication, and benchmarking to improve responsible usage) describes 25 different herd level ABU data collection systems for pig production originating from 15 different European countries [51]. These systems differ in many ways, including the type of collected data (e.g., mg antibiotics used versus days of treatment; kg of biomass versus weight and number of animals at risk), the performed analyses and their respective output (e.g., mg/kg biomass versus treatment incidence). At the same time, they share key components such as data collection, analysis, benchmarking, and reporting. As a result, they face similar challenges for which they need to make the appropriate decision [50]. For example, many herd level data collection systems are used in the framework of a benchmarking scheme to improve (and reduce) ABU and to promote antibiotic stewardship actions. Moreover, once a herd level benchmarking system is available, it is a small step to also develop benchmarking systems for veterinarians. Considering the responsibility of veterinarians for prescribing antibiotics and therefore directly influencing ABU in animals, benchmarking veterinarians is an important option from an antibiotic stewardship perspective [52]. 

**Figure 4:**
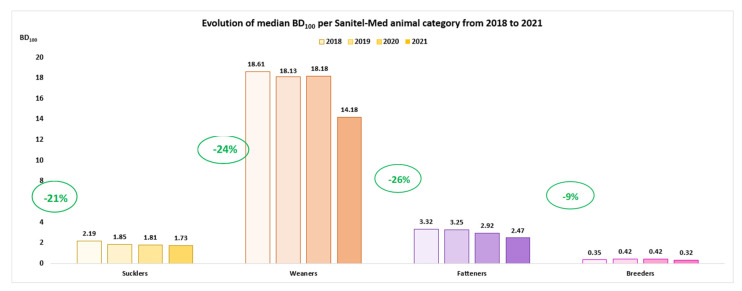
Evolution of the median of the BD_100_ *-distribution in the reference populations for 2018, 2019, 2020, and 2021 of each Sanitel-Med pig category. Zero-use farms per year were excluded for the analysis. (BelVetSac 2021) [52]. * BD_100_ stands for “behandeldagen per 100 dagen” which is the Dutch equivalent of the treatment incidence per 100 days at risk.

Further harmonization of methods and processes, as well as the underlying DDDvet values as called upon by Sanders and Vanderhaeghen [50], could lead to an improved comparability of outcomes and less confusion when interpreting results across systems. Having these type of animal specific data collection systems at farm level is extremely helpful as they allow to describe species-specific evolutions in ABU and assist in creating antimicrobial reduction goals over time both for farmers and veterinarians. As an example, in Belgium, farm level ABU data of the pig sector are being collected since 2014. In agreement with the national sales data, ABU in the pig sector has quite steadily been decreasing over the years. For example, between 2018 and 2021, the data show a decrease of over 15% in the sector-level treatment incidence, distributed over a decrease in the different age categories [52] (Figure 4). These results clearly show differences over the years and between age categories which illustrates the usefulness of farm-level data to gain detailed insight in the situation in the field, allowing for focused and specific approaches.

## 5. Antimicrobial Use in Pig Production in Europe: The Way Forward

### 5.1. Better Health Management and Biosecurity

While the principal role of ABU in food animals should be therapeutic, in reality, use has also been driven by the objective of improving farm productivity and income in the past where poor management and biosecurity practices were sometimes covered up by high ABU. Yet, in current times, this approach is no longer accepted, not by the legislator or the quality insurance schemes, nor by the consumer. Therefore, husbandry systems, production systems, and both management and biosecurity standards should be designed in such a way that the need for antibiotics becomes exceptional or even redundant. 

A total of 116 pig health experts from different European countries were asked to rank ABU alternatives based on a list of defined criteria that assess their expected effectiveness, return of investment, and feasibility [53]. The highest ranked alternative to AMU was an improved biosecurity of the farm, followed by an increased and improved vaccination strategy, the use of zinc (focusing on weaned pigs), the provision of high-quality feed, and the use of improved diagnostics. Aside from the use of zinc, which is banned for medicinal use in Europe since the end of 2021 [17], all the described alternatives are within reach for all pig producers. 

Focusing on farm biosecurity, several studies showed that an improved biosecurity results in a reduced ABU and can maintain similar livestock production levels. A French study in farrow-to-finish herds, showed that farms with lower ABU were associated with the presence of key biosecurity measures (e.g., disinfection of the loading area, all-in/all-out practice) [54]. The Biocheck.UGent® scoring system was used in numerous studies as it enabled researchers to include measurable biosecurity data and directly link them with the ABU data. In Belgium, it was found that farrow-to-finish pig herds with higher internal biosecurity scores used antibiotics for fewer days within one production round, indicating hence a lower ABU [54]. Comparable results were obtained in Germany where the number of sows present at site and a low score for external biosecurity were associated with a higher antibiotic usage [55]. In a study in Finland, it was observed that enhanced external biosecurity levels in large herds co-occurred with lower use of antimicrobials and herds with low biosecurity scores—especially in the internal subcategories—appeared to have higher proportions of resistant isolates [36].

As well as establishing a link between an improved biosecurity and a lower ABU, several management factors were also associated with a reduced ABU in various studies. In a study comprising four European countries, lower ABU was linked to three parameters namely external biosecurity, a higher weaning age (>24 days), and a five-week batch management system [22]. With regard to the benefits of improved internal biosecurity, another study showed a positive link with lower ABU and remarkably a better control of infectious diseases [49]. In Denmark, a study was conducted to identify the management traits of farms that managed to reduce their annual antimicrobial consumption by 10% or more. Several biosecurity-related measures such as cleaning procedures and adequate action regarding diseased animals (e.g., an earlier decision to euthanize) were highlighted by farmers and veterinarians as means to reduce AMU [56]. A Dutch study focused on factors that could play a role in introducing extended spectrum beta-lactamase producing *E. coli* in a farm [57]. It concluded that the higher the levels of biosecurity are (especially the presence of a hygiene lock, and professional pest control), the lower the risk for the farm [57]. An intervention study in Belgium found that improving pig herd management and biosecurity status, in combination with antimicrobial stewardship, helped to reduce AMU in pigs from birth till slaughter by 52%, and in sows by 32% [39]. This “coaching” strategy (i.e., the management and biosecurity interventions) was rather straightforward and led to high implementation rates by the farmers. For example, targeted working habits and routines of the farmer were revised (e.g., changing of needles, hand and personal hygiene, and analysis of water quality). More expensive interventions, such as introducing a new hygiene lock to change clothes/boots and wash hands, were implemented less frequently. 

Addressing the economic benefit of improved biosecurity, the data of the aforementioned study [39] were used by Rojo-Gimeno and showed that, including labor costs of all persons involved (including the coach, veterinarian, and farmer), the participating herds achieved an average financial gain or overall benefit of €2.67 per finisher pig per year from partaking in this “coaching strategy” approach [58]. Another study estimated the economic benefit of improved farm biosecurity to be at €4.46 and €1.23 per sow per year, expressed as the median change in net farm profits among Belgian and French farms respectively [49].

### 5.2. Towards Zero Antimicrobial Use

Raised Without Antibiotics (RWA) is a certification mark that is known in countries such as Denmark [59] and the United States [60]. However, specific inclusion criteria for RWA production and the implementation of RWA in a large number of herds with varying management and housing conditions has only been limitedly investigated. In a recent Belgian study [61], 28 Belgian pig herds were enrolled, and their ABU was followed for a period of 35 months. The goal of the study was to evaluate to what extent pig farms could be coached towards antimicrobial-free pig production and to what extent they could also maintain this status over time. In this study, RWA was defined as no antibiotics from birth until slaughter. ABU had to be generally low on the farm. Group treatments or prophylactic medication with antibiotics were not allowed. Pigs requiring an individual treatment with antibiotics received a special ear-tag and were excluded from the RWA program. The results of the study showed that 13 out of the 28 herds were successfully raising pigs without antibiotics after a coaching period of one year. One year later, still 12 out of the 13 were maintaining this status. Remarkably RWA herds applied less vaccinations, were smaller (median 200 sows, range 85–300) compared to non-RWA herds (median 350 sows, range 180–1250), applied more frequently a 3- and 5-week batch farrowing system, compared to the 4-week system which was used significantly more in non-RWA herds. The weaning age was slightly (not significant) higher on RWA farms (mean 24.9 days) compared to non-RWA farms (mean 23.9 days). This study showed it was possible for farmers to achieve and maintain the RWA status through herd-specific coaching related to prudent ABU and biosecurity.

## 6. Conclusions

Based on the described evolution in ABU in pig production in Europe in the last decade and based on the results already today obtained by the leading producers and countries, it is clear that the use of antibiotics will further diminish and hopefully will become an exceptional act in future pig farming. Obviously, for the majority of the farms both within and beyond Europe, this will require further efforts and focus on better husbandry, biosecurity, and management. In this, it will be key to get everybody involved, including the small-scale breeders and those that are skeptical to change. Ultimately this reduction will also result in the levelling off, and eventually even reversal, of resistance selection, leading to further benefits for animal health as well as human health, global food safety, and food security.

## Data Availability

Not applicable.
